# Tablet of *Spondias mombin* L. Developed from Nebulized Extract Prevents Gastric Ulcers in Mice via Cytoprotective and Antisecretory Effects

**DOI:** 10.3390/molecules26061581

**Published:** 2021-03-12

**Authors:** Maria Elaine Araruna, Pablo Silva, Maria Almeida, Renaly Rêgo, Raiff Dantas, Hilton Albuquerque, Ingrid Cabral, Nadjaele Apolinário, Francinalva Medeiros, Ana Medeiros, Vanda Santos

**Affiliations:** 1 Postgraduate Program in Pharmaceutical Sciences, State University of Paraíba, Campina Grande, PB 58429-500, Brazil; pablo-rayff@hotmail.com (P.S.); cris.freire21@hotmail.com (M.A.); renaly.ivyna@hotmail.com (R.R.); nadjaelemelo@gmail.com (N.A.); francinalvamedeiros@gmail.com (F.M.); anaclaudiamedeiros.uepb@gmail.com (A.M.); 2 Department of Pharmacy, State University of Paraíba, Campina Grande, PB 58429-500, Brazil; raiff.sd@hotmail.com (R.D.); hiltoncesar00@hotmail.com (H.A.); ingridocabral@hotmail.com (I.C.)

**Keywords:** gastric ulcer, *Spondias mombin*, cytoprotection, herbal medicine

## Abstract

*Spondias mombin* L. (Anacardiaceae) has a worldwide distribution and is present in all regions of Brazil. Its leaves, flowers and bark are used as teas in folk medicine to treat diseases of the digestive system. This study aimed to evaluate the acute non-clinical toxicity, gastroprotective activity, and the related mechanisms of action of nebulized extract and tablets based on dried *Spondias mombin* (SmNE). SmNE screening showed the presence of flavonoids (0.65%), polyphenols (25.50%), where the major compound is gallic acid. In the acute oral toxicity assay, a dose of 2000 mg/kg of SmNE administered orally in Swiss mice did not induce any behavioral changes. SmNE (250 or 500 mg/kg p.o) significantly reduced the ulcerative lesion area when compared to the control group in ethanol and non-steroidal anti-inflammatory drug (NSAIDs) models. Results showed that treatment with SmNE (250 mg/kg) reduced acid secretion and gastric content, accompanied with an increase in pH. Previous administration of indomethacin and glibenclamide reversed the protection provided by SmNE, confirming the participation of prostaglandins (PGs) and ATP-sensitive potassium channels (KATP) in its gastroprotective effect. The SmNE tablets met the pharmacopeial quality requirements with gastroprotective activity and similar protection in comparison to the isolated extract administrated. In conclusion, SmNe has a gastroprotective activity related to cytoprotective mechanisms, such as the participation of endogenous prostaglandins and KATP channels, having an anti-secretory effect with systemic action. The formulation obtained presented gastroprotective effects similar to the administration of the extract, the tablets showed favorable compression characteristics by the direct route and met the pharmacopeial quality requirements.

## 1. Introduction

Peptic ulcers are pathologically defined as damage to the mucosal wall, which can reach from the muscle layer to deeper regions, affecting components of the epithelial and connective tissue, including subepithelial myofibroblasts, smooth muscle cells, vessels, and nerves [1].

Among the main types of ulcers, the gastric one stands out as being the most prevalent in the digestive tract, affecting about 10% of the world’s population. It is considered an expanding disease due to current lifestyle trends that include excessive consumption of alcohol and non-steroidal anti-inflammatory drugs (NSAIDs), contamination by *Helicobacter pylori*, and submission to stress conditions that corroborate with increased secretion of hydrochloric acid and pepsin [2,3].

The main symptoms resulting from gastric ulcers are epigastric discomfort, nausea, vomiting, flatulence, which can progress to hemorrhagic damage, thus it is necessary to implement pharmacological and non-pharmacological therapies as treatment options [1]. The main drugs used for treatment act by modulating the action of the aggressors and neutralizing the acidity of gastric secretion, for example, antacids, antagonists of the H_2_ receptors of histamine, and inhibitors of the proton pump. Others induce cytoprotective mechanisms that stimulate the protection of the mucosa, such as sucralfate and prostaglandin analogs [4,5].

These commonly used drugs, although effective, may be related to a series of adverse effects, such as impaired absorption of nutrients, vitamins and minerals, microorganism infections, and in more severe cases, potential profound acid suppression syndromes, in which excessive production of gastrin may be related to hyperplasia of parietal cells [6,7]. Therefore, it is necessary to search for new active substances with gastroprotective activities, and medicines from medicinal plants are emerging as promising alternatives in the prevention and treatment of many diseases, with a lower incidence of side effects when compared to synthetic drugs [8,9,10].

*Spondias mombin* L. belongs to the Anacardiaceae family, popularly known as cajazeira, from Central America and can be found in all regions of Brazil. The leaves, flowers, and bark of this plant are widely used in folk medicine to treat diseases of the digestive system, rheumatism, angina, fever, pain, respiratory diseases, and infectious diseases, among others [9,11,12].

For the development of herbal medicine, it is necessary to incorporate it in a pharmaceutical form, consisting of both active components and vehicles, excipients, and pharmaceutical adjuvants. The choice of the most appropriate pharmaceutical form for herbal medicine should take into account aspects such as the route of administration, its safe use, circumvent stability problems, and how to increase the level of adherence to treatment [13]. The oral route is the most accepted for drug administration due to convenience and systemic performance. Pills are widely used in therapy as they allow for administration of an exact and single-dose—which minimizes errors—and because are economical [14].

Therefore, the objective of this work was to investigate the gastroprotective activity and the possible mechanisms of action of an extract of *S. mombin* and a herbal medicine, developed in the form of tablets, as well as to evaluate the safety of its use in animal models.

## 2. Materials and Methods

### 2.1. Plant and Extract Preparation

The leaves of the species *Spondias mombin* were collected in the municipality of Pombal, Paraíba, Brazil (06°46′13″ S, 37°48′06″ W). The exsiccata was prepared and identified in the herbarium Professor Jayme Coelho de Morais, from the Federal University of Paraíba, under the number EAN-100493; registered in the National Management System for Genetic Heritage and Associated Traditional Knowledge (SisGen) as number A78C788. 

The fresh leaves were cleaned and placed in an air circulation oven at 40 °C for drying. The leaf extract was obtained by extraction via exhaustion in an ultrasound bath with water: ethanol (30:70, *v*/*v*), the plant material was placed in a flask, the solvent was added at a rate of 20 g to 100 mL of solvent and subjected to extraction until complete extraction of its constituents, observed by the solvent’s clarity. After the extraction was completed, the extract was subjected to a concentration process using a rotary evaporator, obtaining a concentration of about 50% of the solvent. Then, the extract was subjected to spray drying in a spray dryer with an inlet temperature of 140 °C and a flow rate of 3 mL/min. The drying aid was colloidal silicon dioxide. The nebulized *S. mombin* extract (SmNE) was obtained.

### 2.2. Phytochemical Screening

To determine the total polyphenol content, the method described by Chandra and Mejia [15] was used. Thus, 1 mL of the SmNE aqueous solution was added to 1 mL of the Folin–Ciocalteau 1N reagent, and this mixture remained at rest for 2 min. Then, 2 mL of a 20% (*w*/*v*) aqueous solution of Na_2_CO_3_ was added, and the mixture remained at rest for another 10 min. Then, the absorbance was read at 757 nm in a spectrophotometer, against a blank composed of distilled water, Folin–Ciocalteau reagent, and 20% Na_2_CO_3_ solution. Gallic acid (GA) was used as a standard and the results were expressed as a percentage of gallic acid per g of extract. All results presented are means (±SEM) of at least three independent experiments.

The determination of the total flavonoid content followed the method of [16]. To 5 mL of each solution (in methanol) of SmNE, the same volume of a solution (in methanol) of 2% AlCl_3_ (*w*/*v*) was added. The mixture remained at rest for 10 min before the absorbance reading at 415 nm, against a blank composed of the AlCl_3_ solution. Quercetin was used as a standard and the results were expressed as a percentage of quercetin per g of extract. All results presented are means (±SEM) of at least three independent experiments.

The condensed tannin content was quantified using the method of [17], in which 0.5 mL of the SmNE sample was added to 3 mL of a vanillin solution (4% *w*/*v* in methanol); then, 1.5 mL of concentrated HCl (37%) was added. The reaction took place in test tubes, immersed in water at about 22 °C. The reading was done at 500 nm, against a blank composed of the solution of vanillin, HCl, and a solution of 50% (*v*/*v*) ethanol in water. Catechin was used as a standard and the results were expressed as a percentage of catechin per g of extract. All results presented are means (±SEM) of at least three independent experiments.

### 2.3. Identification of Chemical Markers by High-Performance Liquid Chromatography (HPLC)

The chemical marker of the SmNE was identified using a high-performance liquid chromatograph (HPLC) (Dionex) coupled to a diode array detector (DAD), with scanning in the UV–visible region, the wavelength used for detection was 254 and 280 nm. For the analyzes, the standards of gallic acid, caffeic acid, kaempferol, catechin, rutin, and quercetin (Sigma Aldrich^®^, Taufkirchen, Germany) were used.

A Gemini NX C18 (Phenomenex) (250 mm × 4.6 mm, 5 μm) chromatographic column was used at 30 °C. The mobile phase consisted of a mixture of acetic acid at 0.1% (*v*/*v*) (A) and methanol (B) in an elution system, according to the following sequence: 0–3 min —10% B isocratic; 3–10 min—gradient up to 12% B; 10–12 min—gradient up to 90% B; 12–17 min —90% B isocratic; 17–20 min —10% B isocratic. The samples were pre-dissolved in a methanol solution (50% *v*/*v*) for analysis.

### 2.4. Animals

Swiss male and female mice (25–30 g) were obtained from the Laboratory of Pharmacological Tests of the State University of Paraíba (UEPB), acclimated under standard laboratory conditions temperature 23 ± 2 °C and controlled light–dark cycles of 12 h and fed with Purina type food pellets and water ad libitum. The experimental protocols were approved by the Ethics Committee on the Use of Animals (CEUA) of Higher Education and Development Center of Paraíba (CESED—PB) and approved under opinion N° 6101032016.

### 2.5. Acute Toxicity Study

The acute oral toxicity test was performed according to the methodology described in guide 423 of the Organisation for Economic Co-operation and Development OECD guidelines [18]. Groups of 3 female animals were used and each dose was repeated once. The animals in the control group orally received saline solution (0.9%), while the animals in the group treated with SmNE received a dose of 2000 mg/kg solubilized in saline (10 mL/kg) solution by p.o.

Systematic behavioral observations were made at the times of 15 min, 30 min, 1 h, 2 h, 4 h after administration and from then on daily until the 14th day through hippocratic screening, according to Almeida et al. [19]. Parameters such as body mass, water consumption, and feed were observed every 24 h for 14 days. At the end of the experiment, the animals were weighed and anesthetized for cardiac puncture collection for biochemical analysis and then euthanized. The organs (liver, spleen, kidneys, and stomach) were removed, weighed, and evaluated macroscopically.

### 2.6. Evaluation of Gastroprotective Activity and Possible Mechanisms of Action of SmNE

#### 2.6.1. Ethanol-Induced Ulcers

After a 12 h fasting period, the animals were orally treated with saline (10 mL/kg), lansoprazole (30 mg/kg) or SmNE (250 mg/kg) and (500 mg/kg). Sixty minutes after the treatments, absolute ethanol (0.2 mL/animal p.o) was administered [20]. One hour after the administration of the necrotizing agent, the animals were euthanized and the stomachs were removed and opened along the great curvature, to determine the ulcerative lesion index (ILU) index.

#### 2.6.2. Ulcers Induced by Nonsteroidal Anti-Inflammatory (Piroxicam)

According to the protocol of Puscas et al. [21], the animals—after a 12 h fasting period—were treated orally with saline (10 mL/kg), cimetidine (100 mg/kg), or SmNE (250 mg/kg). After thirty minutes, gastric lesions were induced with a subcutaneous injection of piroxicam (30 mg/kg). Four hours after the administration of the NSAID, the animals were euthanized, and their stomachs were removed and opened along the great curvature in order to determine the ulcerative lesion index (ILU).

#### 2.6.3. Determination of the Ulcerative Lesion Index

Ulcerative lesions were macroscopically quantified using a magnifying glass and expressed as an ulcerative lesion index (ILU), according to the number and severity of injuries [22], using the following formula:

(1) $$ILU\;=\;\Sigma \;\left(score\;1\times 1\right)\;+\;\left(score\;2\times 2\right)\;+\;(score\;3\times 3)$$

*Score* 1: hemorrhagic stitches and ulcerations up to 1 mm.
*Score* 2: ulcerations of 2 mm.
*Score* 3: deep ulcerations of 3 mm or more.

#### 2.6.4. Determination of Gastric Acid Secretion

The animals submitted to a 12 h fast were anesthetized with (xylazine/ketamine) and through an incision of about 2 cm in the abdomen, after locating the stomach, the pylorus was ligated with suture [23]. The groups received intraduodenal routes, saline (10 mL/kg), cimetidine (100 mg/kg), or SmNE (250 mg/kg). The abdominal wall was sutured, asepsis of the surgery site was ensured by treatment with iodized alcohol, and the animals were then placed in appropriate boxes. Four hours after surgery, the animals were euthanized and their stomachs were removed after tying the cardia, to prevent loss of secreted material. The mucosa was washed with 3 mL of distilled water, and gastric content was collected in test tubes. The volume of gastric content was measured, weighed and the total acidity was quantified using a digital pH meter.

#### 2.6.5. Assessment of the Involvement of Prostaglandins (PGs)

After a 12 h fasting period, the animals were separated into six groups of six animals. The pathway was blocked in three groups with indomethacin (10 mg/kg p.o, a dose that inhibits prostaglandin synthesis without inducing gastric injury), in the other three groups, saline was administered. One hour after the pre-treatments, saline (10 mL/kg), misoprostol (50 μg/kg), or SmNE (250 mg/kg) were administered to the groups previously treated with indomethacin and the other groups. After one hour, absolute ethanol (0.2 mL/animal, p.o) was administered. Fifty minutes after the administration of ethanol, the animals were euthanized to determine the ILU [24].

#### 2.6.6. Assessment of the Involvement of ATP-Sensitive Potassium Channels (KATP)

Four groups of six animals were separated and subjected to a 12 h fast. Two groups were orally pre-treated with 10 mg/kg of glibenclamide (potassium channel blocker) and the other groups received saline (10 mL/kg, p.o). Thirty minutes later, the groups received the treatments: saline (10 mL/kg, p.o) or SmNE (250 mg/kg, p.o). After one hour, the lesion was induced with absolute ethanol. After 50 min of ethanol administration, the animals were euthanized to determine the ILU [25].

### 2.7. Development of the Tablets

Formulations 1 and 2 proposed for the tablets were made with vegetable active pharmaceutical ingredient (VAPI), at a concentration of 250 mg, and the pharmaceutical excipients: microcrystalline cellulose 101, carboxymethylcellulose, starch glycolate, magnesium stearate, and talc. After determining the proportion of each component in the developed formulations, all ingredients were weighed and sieved in a 2 mm opening mesh and then mixed. The magnesium stearate and talc were sieved in a 1.0 mm mesh and then added to the final homogenized mixture for another three minutes (Table 1).

To verify whether the formulations obtained had adequate properties for compression, the following technological parameters were determined: apparent density, compacted density, angle of rest, Hausner factor (HF), and Carr index (CI).

#### 2.7.1. Technological Parameters

For the determination of apparent densities, compaction, the Hausner factor, Carr index, and densification index, 100 g samples were used, which were placed in a graduated cylinder, to determine the gross volume, as described by [26,27]. After that, the samples were subjected to a series of 500, 750, and 1250 sequential drops, according to the properties of the powders. The tests were performed five times. Thus, with the data obtained, it was possible to perform calculations of apparent density (AD), compaction density (CD), Hausner factor (HF), Carr index (CI), and densification index (DI):

(2) $$\mathrm{AD}\;=\;\frac{m}{V_{B}}$$

(3) $$\mathrm{CD}=\;\frac{m}{V_{C}}$$

(4) $$\mathrm{HF}=\;\frac{D_{C}}{D_{B}}$$

(5) $$\mathrm{CI}=\;\frac{D_{C\;}-{\;D}_{B}}{D_{C}}\;\times \;100$$

(6) $$\mathrm{DI}={\;V}_{10}-{\;V}_{500}$$

#### 2.7.2. Rest Angle

The angle of rest was determined according to the height of a glass funnel, with an opening of 11.3 mm, a height of 14 cm, and a fixed base, as described by Santana et al. One hundred grams of each sample was transferred to the funnel in order to observe the cone formed on graph paper. The tangent of the angle of rest was calculated by the ratio tgα = H/R, where α is the angle of rest, H is the height and R is the radius of the cone. The results were calculated by averaging five determinations. The flow time was measured in seconds and was also determined by averaging five measurements.

#### 2.7.3. Production of Tablets

After the technological parameters were realized, 20 tablets were produced directly, with an average weight of 800 mg, using a bench compressor. Weight control and hardness control were performed during the compression process. At the end of the compression process, a sample of 20 tablets was selected to perform tests of medium weight, hardness, friability, and disintegration time, according to the methodology described in [28].

### 2.8. Tablet Quality Parameters

#### 2.8.1. Average Weight

The analysis of mean weight was performed on an analytical balance with 20 tablets of 1800 mg of the pilot formulation. It was not possible to evaluate the parameters of Formulation 2 (500 mg), as this formulation did not form acceptable tablets.

#### 2.8.2. Hardness

The hardness test was performed with ten tablets—using a digital durometer—to assess the resistance of the tablets to crushing. The specification considers that the tablets have a good hardness when it is above 30 N, that is, 3 kgf [28].

#### 2.8.3. Friability

To assess friability, a sample consisting of 20 tablets was used. These were weighed and placed in a friabilometer, at a rotation of 25 rpm. After the test, the tablets were weighed again, and the weight variation was calculated by averaging three determinations.

#### 2.8.4. Disintegration

In the disintegration test, six tablets were used, which were inserted into the basket apparatus. The samples were sequentially immersed in purified water at 37 °C. During the trial, the time taken for the complete disintegration of the tablets was recorded. The results were calculated by averaging the disintegration time of five determinations

### 2.9. Evaluation of the Gastroprotective Activity of the Tablet Formulation by the Ethanol-Induced Ulcer Model

After a 12 h fast, the animals were orally treated with saline (10 mL/Kg), lansoprazole (30 mg/kg), and the SmNE Formulation 2 tablets (800 mg/kg). Sixty minutes after the treatments, absolute ethanol (0.2 mL/animal p.o) was administered, as described by [20]. One hour after the administration of the necrotizing agent, the animals were euthanized and the stomachs were removed and opened along the great curvature in order to determine the ILU.

### 2.10. Statistical Analysis

The results were expressed as mean ± SEM of the mean and the minimum significance level was *p* < 0.05. The differences between the groups were calculated by analysis of variance (ANOVA), followed by the post-test Dunnett and/or Tukey tests, using the software GraphPad Prisma 6.0, San Diego, CA, USA.

## 3. Results and Discussion

### 3.1. Phytochemical Screening

The phytochemical analysis of the *S. mombin* extract (SmNE) revealed flavonoids (0.65%) and polyphenols (25.50%) as its main constituents. These results differ from those of Maduka et al., 2014, who analyzed the ethanolic extract, infusion, and decoction of the leaves and bark of the stem of *S. mombin*, collected in Nigeria. In their study, the presence of different phytochemicals for the leaves and stem—using different means of extraction—was observed [29]. The leaves showed saponins, alkaloids, and tannins in all extraction media. In a study carried out with the leaf extract, the presence of phenols, hydrolyzable tannins, flavones, flavonoids, leucoanthocyanidins, and saponins was observed [30].

According to [31], nebulized extracts—with the addition of a colloidal silicon dioxide adjuvant demonstrated low levels of polyphenol content degradation and a reduction in antioxidant activity when compared to extracts containing other adjuvants. For this reason, this type of extract was chosen to carry out this work.

### 3.2. High-Performance Liquid Chromatography (HPLC)

**Figure 1:**
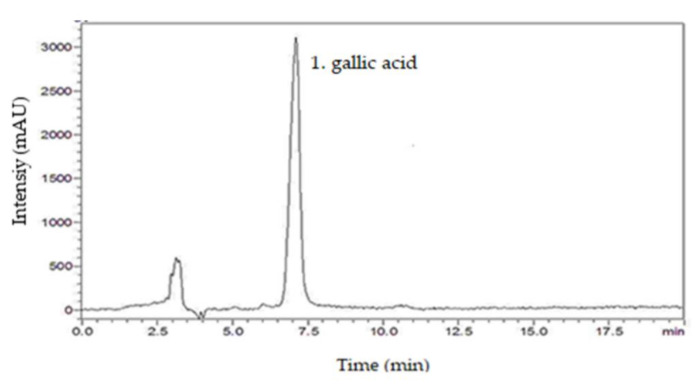
HPLC chromatogram of the nebulized leaf extract of *Spondias mombin*. 1. gallic acid (7 min).

From the chromatographic analysis of SmNE, the presence of the following compounds was investigated: caffeic acid, gallic acid, kaempferol, catechin, rutin, and quercetin. By comparing the retention times of the respective standard samples with the solutions of the extract, it was possible to identify the presence of the compound gallic acid, by using analytical standards based on the similarity between the retention times and the ultraviolet absorption spectrum [32]. The chromatogram of the SmNE at a wavelength of 254 nm highlights the good peak resolution in 7 min, which corresponds to gallic acid (Figure 1).

Therefore, this separation method was considered effective for the identification of the chemical marker of the extract. This substance can be considered as a parameter for quality control, to calculate the final amount of plant extract in the finished tablets.

Gallic acid is a polyphenol found in some plants, fruits, and products of plant origin and can be obtained by acid hydrolysis of tannins. It has proven antimutagenic, anti-inflammatory, and antioxidant activities [33]. Previous studies by [34], demonstrated the presence of gallic acid and ellagic acid in the ethanolic extract of *S. mombin* leaves.

Some polyphenols, such as gallic acid, are antioxidants, that is, they can sequester reactive oxygen species, which cause oxidative damage through lipid peroxidation [35]. The gastroprotective effect of gallic acid in isolation has been described and occurs through the inhibition of mitochondrial apoptosis [36].

### 3.3. Acute Toxicity

During the 14 days following the administration of the extract, there was no death or change in the animals’ behavior, and the extract could be classified in Class 5 of toxicity, according to the Globally Harmonized System (GHS), with an LD50 estimated as greater than 2000 mg/kg. The extract was therefore considered to be of low toxicity. When assessing water and feed consumption, as well as target organ analysis, there was no statistically significant difference between the group treated with the extract and the control group (*p* < 0.05).

These results are in agreement with studies by [34], who demonstrated the safety of using *S. mombin* extracts in rodents. 

### 3.4. Ethanol-Induced Ulcers 

Validation of the gastroprotective activity of a plant product or drug can be done by ulcer induction methods in animal models. Ethanol, when ingested in excess, causes hemorrhage of small vessels in the gastric mucosa and tissue necrosis, which causes loss of integrity of the vascular endothelium and an increase in its permeability. This results in the infiltration of inflammatory cells, inhibition of cell growth factors, stimulation via apoptosis, and lipid peroxidation [37].

**Figure 2:**
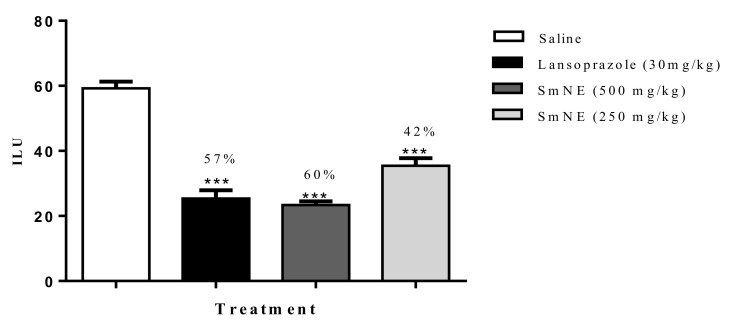
Effect of oral pretreatment with SmNE in the ethanol-induced ulcer model. SmNE administered in doses of 250 and 500 mg/kg p.o, lansoprazole 30 mg/kg. The results are expressed as the mean ± SEM (group). One-way analysis of variance (ANOVA), followed by Dunnett’s multiple comparison test. *** (*p* ˂ 0.001), versus negative control *n* = 6 animals.

The results obtained in the ethanol induction model demonstrated that SmNE, at doses of 250 and 500 mg/kg or lansoprazole (30 mg/kg), inhibited ulcer formation by 42, 60, and 57%, respectively, when compared with the negative control (Figure 2). 

The results obtained in this study show that the extraction method and drying by nebulization do not interfere with the pharmacological activity of the extract. For the other protocols, the dose of 250 mg was standardized, which was effective in the ethanol model, with no significant difference in the dose of 500 mg/kg.

### 3.5. Ulcers Induced by Non-Steroidal Anti-Inflammatory Drugs (NSAIDs)

**Figure 3:**
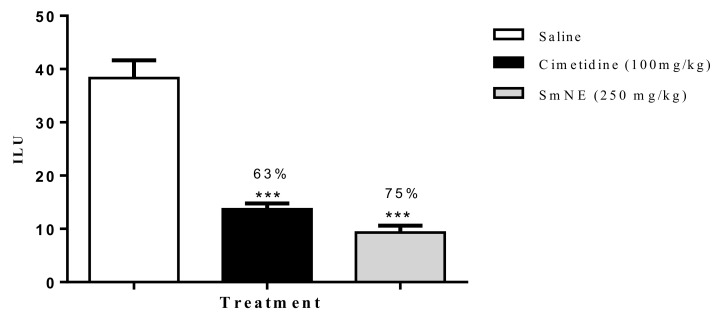
Effect of oral pretreatment with SmNE on the non-steroidal anti-inflammatory drug (NSAID)-induced ulcer model. SmNE administered at a dose of 250 mg/kg p.o, cimetidine 100 mg/kg. The results are expressed as the mean ± SEM (group). One-way analysis of variance (ANOVA), followed by Dunnett’s multiple comparison test. *** (*p* < 0.001) versus negative control *n* = 6 animals.

According to the results obtained in this model, it was observed that SmNE and cimetidine significantly reduced the ILU, by 75 and 63%, respectively, when compared to the negative control (Figure 3).

Non-selective NSAIDs are the first choice drugs for the treatment of pain and inflammation, however, their main adverse effect is the formation of gastric ulcers. Both adverse and therapeutic effects are caused by the inhibition of the cyclooxygenase enzyme with a consequent reduction in PG synthesis. This inhibition—associated with other pathogenic factors, such as oxidative phosphorylation, reduction in cell proliferation of the mucosa, and activation of neutrophils—followed by increased endothelial adhesion, causes the occlusion of microvessels and an increase in oxygen-reactive metabolites. This induces oxidative tissue damage, which seems to play an important role in the pathophysiology of NSAID-induced ulceration [38].

Prostaglandins E2 and I2 act in the synthesis of mucus and bicarbonate, in the regulation of acid secretion and blood flow of the gastric mucosa, and their inhibition critically compromises gastric cytoprotection [39]. NSAIDs, such as piroxicam, can produce gastric lesions in humans and experimental animals.

It has been proposed that administration of NSAIDs also reduces the levels of adenosine triphosphate (ATP), causing a reduction in cellular energy in response to mitochondrial damage, which is the first event in topical mucosal erosions. Cells with defective mitochondrial function, possibly due to changes in antioxidant activity and formation of reactive species, are exposed to ulceration [40].

These results suggest that the gastroprotective effect is promoted by cytoprotective mechanisms of the mucosa, since gastric lesions induced by NSAIDs involve the inhibition of these mediators. These obtained results corroborate the study by [9], in which *S. mombin* extract significantly inhibited ulcerative lesions.

### 3.6. Evaluation of Antisecretory Activity

In this model, 4 h after pyloric ligation, intraduodenally administered SmNE significantly reduced the volume and weight of gastric content concerning the saline. Cimetidine, a known H_2_ receptor antagonist, also showed a significant reduction concerning the saline. In terms of the gastric pH, SmNE increased in relation to the vehicle, a result similar to that obtained by cimetidine (Table 2).

The acute pyloric ligation proposed by Shay et al. [23], aims to increase both the production of histamine and the total gastric content. The peak rise in gastric cell histamine content occurs 4 h after ligation, returning to normal levels 24 h later. This period of peak elevation corresponds to observed increases in the volume and acidity of gastric secretion. The accumulation of acid and pepsin in this model leads to automatic digestion of the gastric mucosa [41,42].

The increase in secretion potentiates the actions of acid and pepsin, and the protective capacity of the mucosal defense barrier depends on the quality and quantity of secreted mucus, both of which are dependent on prostaglandins. The gastroprotective activity of the drug or extract tested can occur by reducing gastric acid secretion and/or increasing mucus secretion in this experimental model [43,44].

Intraduodenal administration is important to investigate the activity of the extract systemically, avoiding direct contact of the substance with the gastric mucosa. When administered intraduodenally, SmNE showed antisecretory activity, in which a reduction in the volume and weight of gastric content and an increase in pH can be observed, suggesting that antisecretory activity may contribute to the gastroprotection conferred to the extract. The results obtained corroborate those obtained by [34], in which the juice of the fruits of *S. mombin*—which is a 100% solution—also showed anti-secretory activity. 

### 3.7. Evaluation of the Role of Prostaglandins (PG’s) in the Gastroprotective Effect

**Figure 4:**
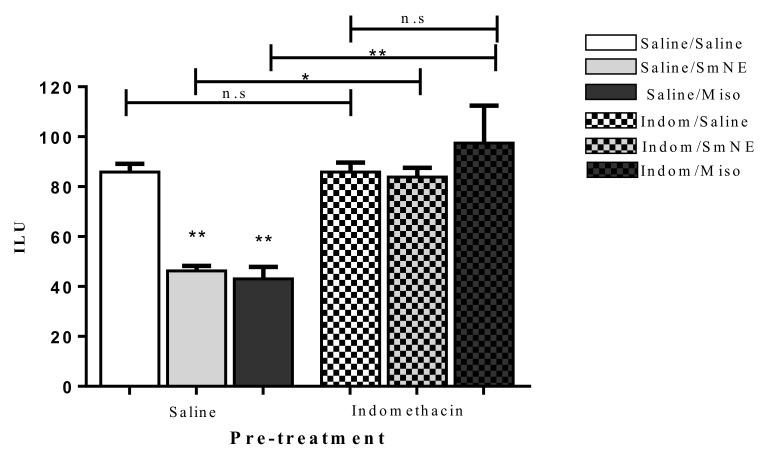
Oral pretreatment effect with SmNE in the ethanol-induced ulcer model, with pretreatment with indomethacin (10 mg/kg). SmNE at a dose of (250 mg/kg), misoprostol (50 µg/kg). The results are expressed as the mean ± SEM (group). One-way analysis of variance (ANOVA), followed by Tukey’s multiple comparison test. * (*p* < 0.05), ** (*p* < 0.01) versus negative control *n* = 6 animals. Saline, SmNE, and misoprostol groups pretreated with saline versus pretreatment with indomethacin * (*p* ˂ 0.05), ** (*p* < 0.01). *n* = 6 animals. n.s—means not significant.

Previous administration of SmNE and misoprostol (analog of PGE1) in different groups, significantly reduced the incidence of the gastric ulcer index when compared to the negative control. This effect was reversed in the groups in which PG synthesis blockade was performed with previous administration of indomethacin (10 mg/kg) p.o (Figure 4). 

Indomethacin is a non-selective cyclooxygenase (COX) inhibitor that, by suppressing the constitutive COX-1 activity expressed in the gastrointestinal tract, compromises the integrity of the mucosa. This occurs due to the inhibition of the cytoprotective functions of endogenous PGs against the aggressor stimulus, compromising the mucosal blood flow, mucus and bicarbonate secretion, and inhibition of gastric acid secretion, while also preventing the activation and adhesion of neutrophils that defend the epithelial cells of the gastrointestinal tract [45,46].

The results show that in the presence of indomethacin, there is no significant difference between the groups treated with SmNE or misoprostol compared to those treated with saline. These data indicate that the gastroprotective effect is dependent on prostaglandin synthesis as there is a significant increase in the number of ethanol-induced ulcerations when this pathway is blocked.

According to [47], compounds capable of inducing or positively modulating the constitutive prostaglandins (PGE2 and PGI2) can improve the regeneration of gastric epithelial cells by minimizing the action of aggressive factors in addition to acting in several protective mechanisms.

### 3.8. Evaluation Involving ATP-Sensitive Potassium Channels in the Gastroprotective Effect

**Figure 5:**
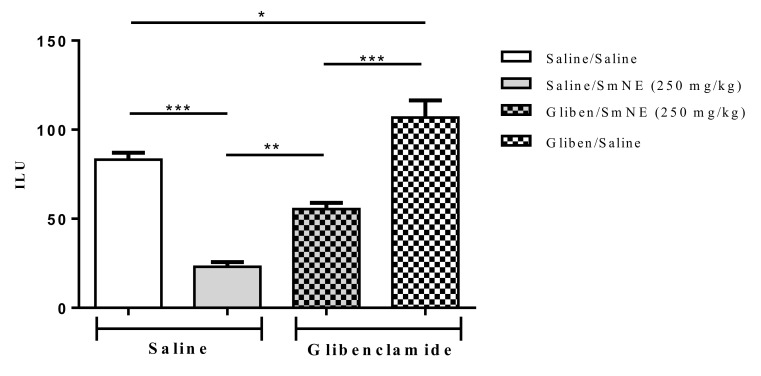
Effect of oral pretreatment with SmNE in the ethanol-induced ulcer model and pretreatment with glibenclamide (10 mg/kg). SmNE at a dose of (250 mg/kg). The results are expressed as the mean ± SEM (group). One-way analysis of variance (ANOVA), followed by Tukey’s multiple comparison test. * (*p* < 0.05), *** (*p* < 0.01). Saline and SmNE groups pretreated with saline versus pretreatment with glibenclamide * (*p* < 0.05), ** (*p* ˂ 0.01), *** (*p* < 0.001). *n* = 6 animals.

The results show that previous administration of glibenclamide, an ATP-sensitive potassium channel blocker, partially reversed SmNE gastroprotection in ethanol-induced lesions. These results indicate that the effect promoted by the extract is at least partly dependent on the activity of these channels (Figure 5).

ATP-sensitive potassium channels (KATP) have been identified in various tissues, such as the pancreas, heart, liver, and stomach, where they regulate the flow of potassium in the cell membrane [48,49,50].

Activation of the KATP channels can occur via two main ways: via the NO/cGMP/KATP pathway, where NO activates soluble guanylate cyclase and increases levels of cyclic guanosine monophosphate (cGMP) which will ultimately activate KATP [51]; and also by the activity of endogenous PGs, which—when activating EP3 receptors—stimulate KATP, [4,25,52,53]. Thus, it is believed that activation of KATPs is related to contractility, regulation of blood flow, and gastric acid secretion.

Studies by [54], when co-administering glibenclamide, observed a loss of the gastroprotective effect of its test compound, which significantly increased the ulcer index. This effect was accompanied by increased levels of TNF-α and NF-κB in the gastric mucosa. These clues suggest that these targets are important protective factors.

Therefore, the results show that blockage of KATP channels caused by previous administration of glibenclamide significantly reversed the effect of SmNE in the inhibition of gastric lesions induced by ethanol. Knowing that the SmNE effect involves the participation of prostaglandins, it can be considered that endogenous prostaglandins act as activators of the KATP channels as part of the gastroprotective effect of the extract [52].

### 3.9. Formulation Development

The use of the nebulized extract was favored for obtaining the proposed formulation due to the ease of handling, solubility, and mixing with the excipients.

The SmNE concentration used in the development of the formulations was determined in a pharmacological study, using the ethanol induction model. The selected pharmaceutical excipients and proportions used are described in Table 1.

The proposed formulations showed poor flow properties (in the evaluation of the technological parameters of apparent density), compacted density, angle of rest, Hausner’s factor (PH), and Carr’s index (IC) in both lots (Table 3).

The extracts produced with colloidal silicon dioxide had better flow characteristics and a reduced tendency to agglomerate compared to other adjuvants. However, the high load of the plant constituents and reduced particle sizes could cause flow problems, which could lead to unfavorable behavior in conditions of environmental humidity [55].

It is worth pointing out that the flow properties of dry powders have a direct relationship with their behavior during storage, handling, and processing. For the direct compression process, the VAPI must have a good flow capacity [26]. According to studies by Bushra et al. [56], the use of microcrystalline cellulose as a diluent for the direct compression method showed excellent compressibility and its use together with a lubricant provides a good flow of the product through the compressor feeder. The higher proportion of microcrystalline cellulose contained in Formulation 2 optimized the flow properties (Table 3).

**Figure 6:**
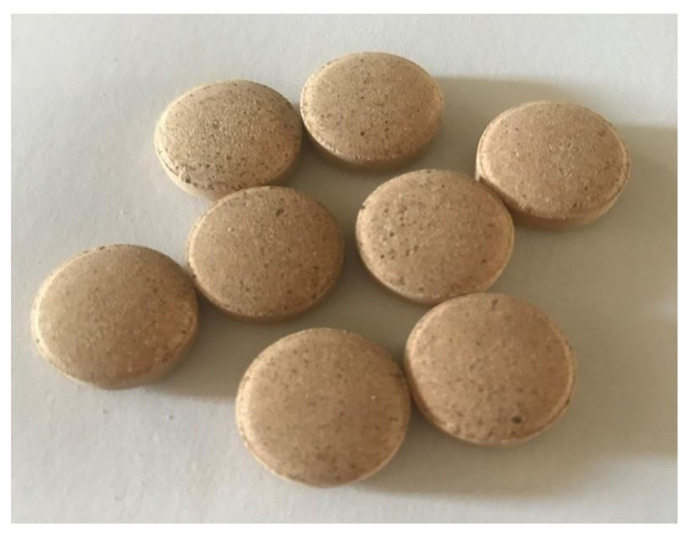
Image of tablets.

The tablets showed a characteristic color of the extract and an absence of grooves, with a diameter of 14 mm and height of 3 mm. The tablets showed no roughness or imperfections to the naked eye, while the edges did not appear to have a tendency to wear away (Figure 6). 

### 3.10. Evaluation of the Quality Parameters of the Pills (P2)

#### 3.10.1. Determination of the Average Weight of the Tablets

**Figure 7:**
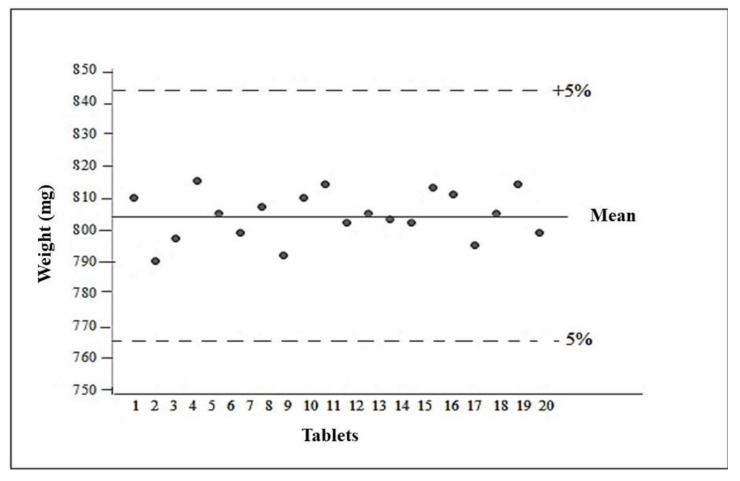
Average tablet weight.

The average weight of the formulation analyzed was 804 ± 0.00728 mg. After obtaining the average weight, the weight variation was calculated, with the upper limit 5% above the average and the lower limit 5% below the average, so that they should be between 760 and 840 mg. All tablets were within the designated limits of acceptance, complying with the specifications described in United States Pharmacopeia 2013 [28] (Figure 7).

The evaluation of the strength parameters of tablets is important, as they must be resistant so that they do not break during manufacturing or storage operations.

#### 3.10.2. Determination of Hardness

The tablets produced had an average hardness of 35.72N (Table 4). The United States Pharmacopeia 2013 [28] considers values above 30 N to represent good hardness, that is, 3 kgf, which shows that the values obtained meet the hardness specification. Regarding this parameter, caution is necessary, as tablets with high hardness can alter the product’s disintegration.

#### 3.10.3. Assessment of Friability

The difference between the initial and final weight, after removing residues from the tablets represents friability. Loss of less than 1.5% of the total weight of the tablets is considered acceptable. The calculated friability for the tablets was 0.13% (Table 4), which is also in line with the limit determined by [28].

#### 3.10.4. Determination of Disintegration Time

For the release of VAPI to occur, it is necessary to disintegrate the tablet in the gastrointestinal tract, because if it is inadequate, it will limit the dissolution and absorption of VAPI. The time limit established as a general criterion for the disintegration of uncoated tablets is 30 min unless otherwise indicated in the individual monograph [57]. The tested pills met the requirements, the tested pills disintegrated completely in 6:15 min (Table 4). 

### 3.11. Evaluation of the Gastroprotective Activity of the Tablet Formulation in the Ethanol-Induced Ulcer Model

**Figure 8:**
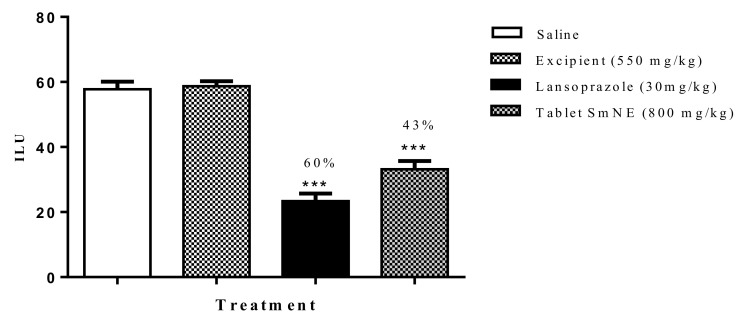
Effect of oral pretreatment with the SmNE tablet on the ethanol-induced ulcer model. SmNE tablet 800 mg/kg p.o, lansoprazole 30 mg/kg, and excipients 550 mg/kg. The results are expressed as the mean ± SEM (group). One-way analysis of variance (ANOVA), followed by Dunnett’s multiple comparison test. *** (*p* < 0.001) versus negative control *n* = 6 animals.

To assess whether the processing of the pharmaceutical form interfered with the pharmacological properties of the extract, the ethanol induction protocol was performed. The results obtained show that the formulation (800 mg/kg) and lansoprazole (30 mg/kg) inhibited gastric lesions induced by ethanol by 54 and 60%, respectively, when compared to the negative control. Excipients alone did not inhibit ulcerations compared to saline (Figure 8).

The tablets work satisfactorily, preserving the properties of the pure extract. In studies carried out with extracts of Maytenus aquifolium Martius dried by nebulization, the extracts showed significant antiulcerogenic activity in rats, thus, proving that the spray drying process did not cause changes in the biological activity of the plant [31,58].

## 4. Conclusions 

The results of the present investigation indicate that SmNE has a gastroprotective activity that is related to cytoprotective mechanisms, such as the participation of endogenous prostaglandins and KATP channels. In addition to this, SmNE has an anti-secretory effect with systemic action. The formulation obtained here presented a gastroprotective activity similar to that produced by the administration of the extract. The tablets showed favorable compression characteristics via the direct route and met the pharmacopeial quality requirements, which guarantees the stability and effectiveness of the final product.

## 5. Patents

The privilege of innovation. Registration number: BR 10 2017 023586 6, title: method and pharmaceutical formulation with gastroprotective activity from a nebulized extract of *Spondias mombin*. Registration institution: INPI—National Institute of Industrial Property. Deposit: 11/01/2017.

## Figures and Tables

**Table 1:** Raw materials, function, and proportion used in the tested formulations. Note: VAPI: Vegetable Active Pharmaceutical Ingredient.

Raw Materials	Function	Formulation 1	Formulation 2
Nebulized extract of *Spondias mombin* (SmNE)	VAPI	250 mg	250 mg
Microcrystalline Cellulose 101	Diluent	155 mg	452 mg
Carboxymethylcellulose	Binder	10 mg	10 mg
Glycolate starch	Disintegrating	10 mg	16 mg
Magnesium stearate	Lubricant	25 mg	24 mg
Talc	Lubricant	50 mg	48 mg

**Table 2:** Effect of oral SmNE administration on gastric content parameters after pyloric ligation. The results are expressed as the mean ± SEM (*n* = 6/group). Unidirectional ANOVA, followed by Dunnett’s multiple comparison test. * (*p* ˂ 0.05), ** (*p* ˂ 0.01) versus negative control. *n* = 6 animals.

Treatment (p.o)	pH	Gastric Volume (mL)	Gastric Content (mg)
Saline (10 mL/kg)	3.3 ± 0.8	0.6 ± 0.1	593 ± 234.
Lansoprazole (30 mg/kg)	4.7 ± 0.6 **	0.12 ± 0.08 **	235 ± 78 **
SmNE (250 mg/kg)	4.5 ± 0.5 *	0.28 ± 0.08 *	276 ± 98 *

**Table 3:** Results of flow properties. Note: Angle of rest of 36–40° = acceptable flow; 41–45° = passable flow; from 46 to 55° = poor flow. CI from 16 to 20% = weak flow; CI of 21 to 31% = poor flow. PH between 1.26 and 1.45 = poor flow; PH between 1.35 and 1.45 = poor flow (cohesive powders).

Properties	Formulation 1	Formulation 2
Apparently density (g/cm^3^)	0.35	0.47
Compaction density (g/cm^3^)	0.43	0.58
Resting angle (α)	41.00	36.00
Carr Index (CI%)	25.00	20.00
Hausner index (PH)	1.38	1.25

**Table 4:** Characterization data for the batch of tablets.

Parameter	Result	Specification
Average weight (mg)	804	764.10–844.62
Hardness (N)	35.72	>30 N
Friability (%)	0.13	˂1.5%
Disintegration time (m)	6:15	<30 min

## Data Availability

The data presented in this study are available on request from the corresponding author.

## Sample Availability

Samples of the compounds are not available from the authors.
